# MicroRNA-495 suppresses cell proliferation and invasion of hepatocellular carcinoma by directly targeting insulin-like growth factor receptor-1

**DOI:** 10.3892/etm.2022.11432

**Published:** 2022-06-09

**Authors:** Ying Ye, Juhua Zhuang, Guangdong Wang, Saifei He, Suiliang Zhang, Guoyu Wang, Jing Ni, Jiening Wang, Wei Xia

Exp Ther Med 15:1150–1158, 2018; DOI: 10.3892/etm.2017.5467

Following the publication of this paper, it was drawn to the Editors’ attention by a concerned reader that certain of the cell migration and invasion assay data shown in Fig. 5C were strikingly similar to data appearing in different form in other articles by different authors. Owing to the fact that the contentious data in the above article had already been published elsewhere, or were already under consideration for publication, prior to its submission to *Experimental and Therapeutic Medicine*, the Editor has decided that this paper should be retracted from the Journal. The authors were asked for an explanation to account for these concerns, but the Editorial Office did not receive a reply. The Editor apologizes to the readership for any inconvenience caused.

